# Benefits of Dynamic Nomogram Models for Elderly Diffuse Large B-Cell Lymphoma Patients' Early Death Prediction and Clinical Application

**DOI:** 10.1155/2023/7874239

**Published:** 2023-04-15

**Authors:** Lingke Zhang, Hongmei Jing, Shuhan Tang, Jing Wang, Ping Yang

**Affiliations:** Department of Hematology, Peking University Third Hospital, Beijing 100191, China

## Abstract

**Background:**

Diffuse large B-cell lymphoma (DLBCL) is an aggressive malignancy, and about 60% of the patients are diagnosed in their elderly age (≥65 years old). However, little is known about the early mortality and risk factors related to elderly patients with DLBCL. *Methodology*. From 2000 to 2019, elderly patients diagnosed with DLBCL in the Surveillance, Epidemiology, and End Result (SEER) database were involved in this research and served as test cohort. Moreover, elderly DLBCL patients from Peking University Third Hospital were used for external validation cohort. Risk factors were identified by univariate and multivariate logistic regression analyses. Nomogram models were constructed based on significance risk factors to predict the overall and cancer-specific early death. Besides that, the predictive value of the models was validated by receiver operating characteristic (ROC) analysis. Calibration plots were used to evaluate the calibrating ability. Clinical benefits of nomogram were evaluated by decision curve analysis (DCA).

**Results:**

15242 elderly DLBCL patients obtained from the SEER database and 152 patients from Peking University Third Hospital were enrolled in this research. In the SEER database, 36.6% (5584/15242) of the patients had early death and 30.7% (4680/15242) of them were cancer-specific early death. Marital status, Ann Arbor stage, surgical treatment, radiotherapy, and chemotherapy were significant risk factors for overall and cancer-specific early death of elderly DLBCL patients. Nomograms were constructed according to these risk factors. Then, ROC analysis showed that the AUC of OS was 0.764 (0.756~0.772), and CSS was 0.742 (0.733~0.751). In the validation group, the AUC of OS was 0.767 (0.689~0.846) and CSS was 0.742 (0.743~0.83).

**Conclusion:**

The calibration plots and DCA analysis revealed that the nomograms were good at early death prediction and clinical application. Predictive dynamic nomogram models for elderly DLBCL patients were established and validated, which might play an essential role in helping physicians enact better treatment strategies.

## 1. Introduction

Diffuse large B-cell lymphoma (DLBCL) is one of the most common subtypes of non-Hodgkin lymphoma (NHL), which accounts for approximately 30% to 40% of patients with NHL [1, 2]. DLBCL is known as an aggressive carcinoma, with the features including rapidly growing cancers in the liver, bone marrow, lymph nodes, and other organs [3]. As the wide application of radiotherapy, chemotherapy, and immunotherapy combined with autologous stem cell transplantation, the survival of DLBCL patients improves dramatically and DLBCL is found to be a curable carcinoma [4]. Such as it is, there are still about 30%~40% DLBCL patients failing to achieve remission or relapsing, which contributes to the morbidity and mortality of DLBCL patients [5, 6]. Moreover, about 60% of the DLBCL patients are diagnosed at the age of ≥65 years old [7], and it is found that the risk of death significantly increases with age [8, 9]. However, there is little research focused on the early death of elderly DLBCL patients.

DLBCL has a wide range of prognoses in the worldwide population. The identification of risk factors and prognosis prediction is particularly vital for health care and making treatment decisions. Early death is defined as survival time less than 3 months, and the factors for early death of patients with DLBCL are still unknown. Nomogram is a type of prognostic model combined with risk factors to predict outcomes of patients, which has obtained a lot of attention in the oncology field [10–13]. Besides that, dynamic nomogram is useful for explaining the heterogeneity in the outcomes of elderly DLBCL patients with diverse clinical characteristics, providing individual prediction for the probability of outcome events. Therefore, the aim of this research was to identify risk factors, meanwhile constructing dynamic nomograms to predict the overall and cancer-specific early death of elderly DLBCL patients.

## 2. Methods

### 2.1. Patients

In this research, data of elderly (≥65 years old) patients with DLBCL from the dataset “incidence SEER research plus data 17 registries, Nov 2021 Sub (2000-2019)” was extracted from the Surveillance, Epidemiology, and End Results (SEER) database, which is a public database that contains about 27.8% of the US cancer patients [14]. Histology codes from the third edition of the International Classification of Diseases for Oncology (ICD-O-3) including 9680/3, 9684/3, and 9688/3 were used to obtain the interesting cohort. All patients included in this research were confirmed as positive histology, who had one primary tumor only and completed clinical information. Therefore, a total of 15242 DLBCL patients from the SEER database were included in this research. Moreover, 152 elderly DLBCL patients from Peking University Third Hospital were utilized as external validation cohort. Early death was defined as survival time less than 3 months after diagnosis. This research was approved by the ethics committee of Peking University Third Hospital. The ethics committee approval number is M2021034.

### 2.2. Statistical Analysis

Categorized data was described by numbers and percentages (*N* and %). Univariate and multivariate logistic analyses were utilized to identify the risk factors associated with OS and CSS early death of elderly DLBCL patients. Discrimination was judged by the receiver operating characteristic (ROC) curve; the higher the area under the curve (AUC) was, the better the accuracy of the nomogram would be (13). Calibration plots were used to evaluate calibrating ability. Moreover, the clinical benefits of nomogram were evaluated by decision curve analysis (DCA). All analyses were performed by R software (4.1.3). *P* value < 0.05 (two-tail) was regarded as statistically significant.

## 3. Results

### 3.1. Demographic and Clinical Characteristics of Elderly Patients with DLBCL

The demographic and clinicopathological characteristics of elderly patients with DLBCL from the SEER database (*n* = 15242) and external validation cohort (*n* = 152) are shown in Table 1. From the SEER database, most of the patients were white (86.1%), while the black, AI/AN patients, and API patients accounted for 4.3%, 0.5%, and 9.1%, respectively. The gender distributions were not different. The most common Ann Arbor stage was IV (34.7%), followed by stages I (25.1%), II (17.8%), and III (16.9%). About two-thirds of the patients was nodal (64.2%). Compared with chemotherapy (64.1%), few of the patients receipted surgical treatment (27.7%) or radiation (15.8%). There was no significant difference between the SEER cohort and the external validation cohort.

### 3.2. Mortality of Early Death of Elderly Patients with DLBCL

As shown in Table 2, 36.6% (5584/15242) of the patients suffered from early death and 30.7% (4680/15242) of them were cancer-specific early death. Unmarried patients had slightly higher early mortality than married patients. White patients had the highest early (86.0%) and cancer-specific (85.8%) mortality, followed by API, black, and AI/AN patients. Mortality rates increased with household income and peaked at the median household income of $55,000–$69,999. Tumor site at nodal caused a higher early mortality compared with extranodal. The highest early mortality was shown in Ann Arbor stage IV. Moreover, patients receipted surgical treatment, radiotherapy, or chemotherapy had lower early mortality compared with those not performed. There were no significant differences in the sex group.

### 3.3. Risk Factors Associated with Early Death of Elderly DLBCL Patients

Risk factors of early death of elderly DLBCL patients were identified by univariate and multivariate logistic regressions. As displayed in Tables 3 and 4, variables including marital status (OR: 1.1, 95% CI: 1.02-1.18), Ann Arbor stage, surgical treatment (OR: 0.67, 95% CI: 0.61-0.73), radiotherapy (OR: 0.33, 95% CI: 0.29-0.37), and chemotherapy (OR: 0.14, 95% CI: 0.13-0.15) were significantly associated with the probability of overall early death of elderly DLBCL patients. Moreover, multivariate logistic regression analysis showed that unmarried status, advanced Ann Arbor stage, no surgical treatment, no radiotherapy, and no chemotherapy were significant risk factors for cancer-specific early death of elderly DLBCL patients, all of which were *P* < 0.05.

### 3.4. Dynamic Nomogram Construction

**Figure 1:**
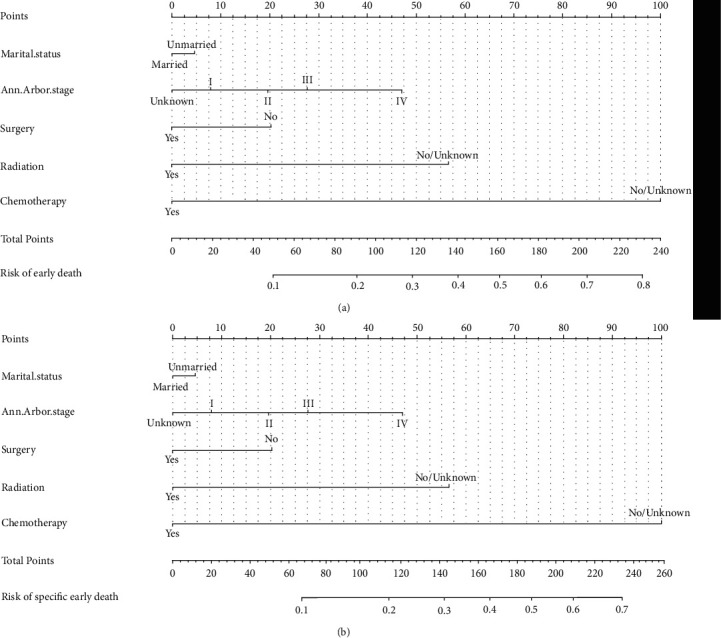
The predictive nomogram for the overall early death (a) and specific early death (b) for DLBCL patients.

Risk prediction nomogram of the SEER cohort was constructed according to the multivariate logistic regression analysis results. The odds of early death among elderly DLBCL patients could be predicted by calculating the scores of each factor. As shown in Figure 1, Ann Arbor stage, surgical treatment, radiotherapy, and chemotherapy were great predictors for elderly DLBCL patients' overall and cancer-specific early death in the nomogram prediction models. To obtain the dynamic nomogram for OS, visit https://zhanglingbao.shinyapps.io/DynNomapp/, and dynamic nomogram for CSS, visit https://zhanglingbao.shinyapps.io/DynNomapp_CSS/.

### 3.5. Nomogram Validation

**Figure 2:**
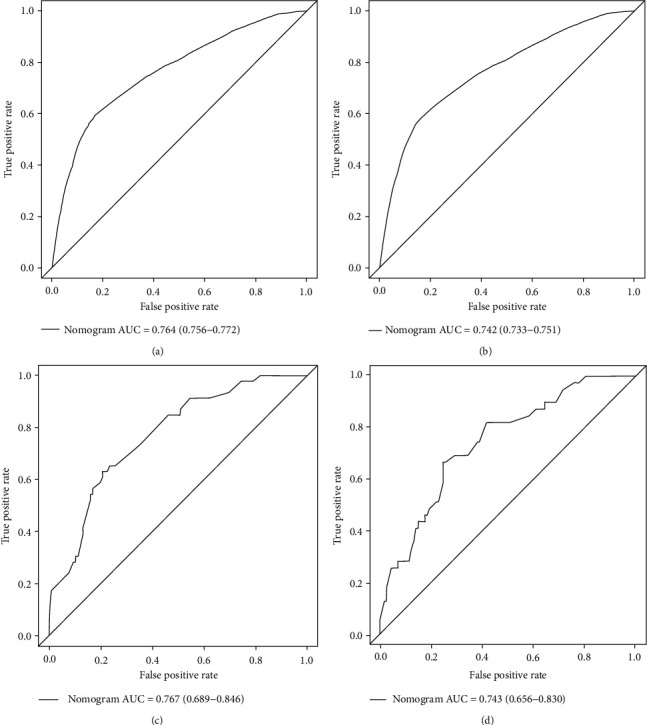
The ROC curves for the nomogram of overall early death (a) and specific early death (b) in the SEER database. The ROC curves for the nomogram of overall early death (c) and specific early death nomogram (d) in the validation cohort.

**Figure 3:**
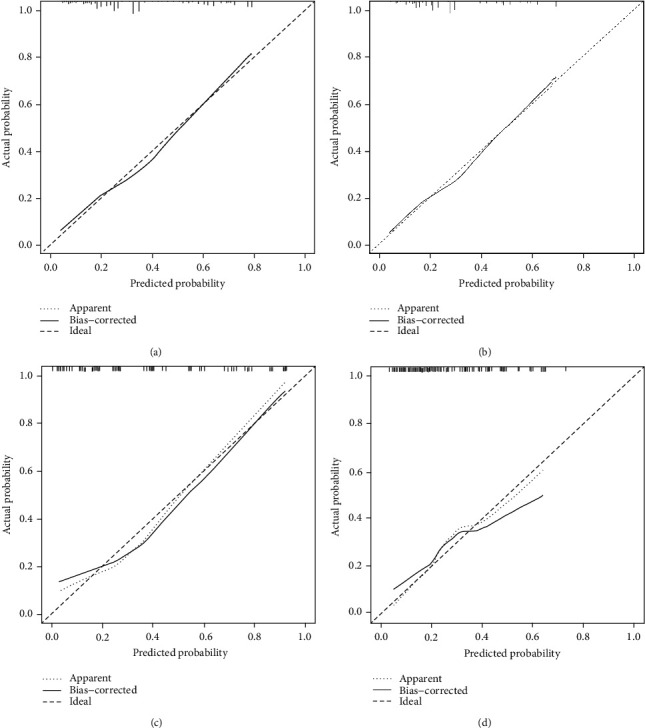
The calibration plots for the nomogram of overall early death (a) and specific early death (b) in the SEER database. The calibration plots for the nomogram of overall early death (c) and specific early death nomogram (d) in the validation cohort.

**Figure 4:**
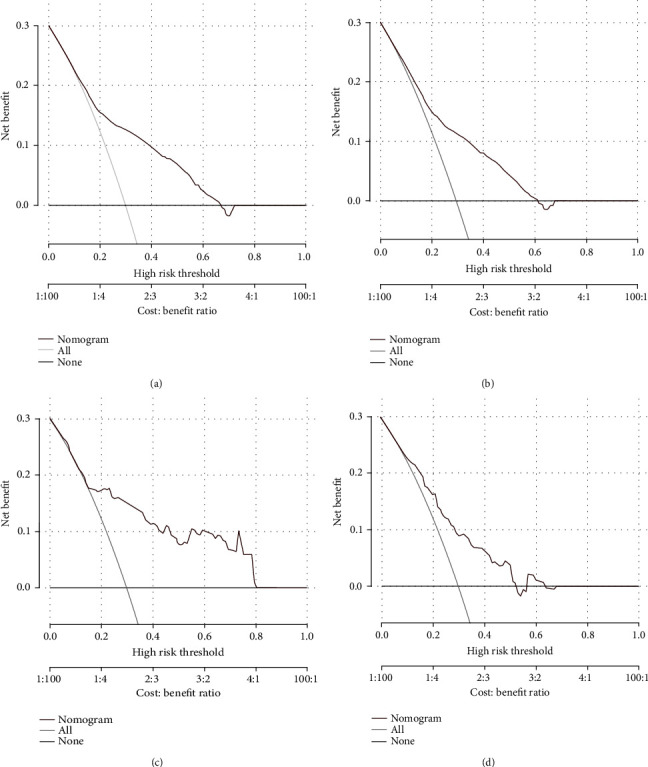
The decision curve analysis (DCA) for the nomogram of overall early death (a) and specific early death (b) in the SEER database. The decision curve analysis (DCA) for the nomogram of overall early death (c) and specific early death nomogram (d) in the validation cohort.

The ROC, DCA analysis, and calibration plots were utilized to detect prediction efficiency on the probability of early death, and the results showed that nomograms had a good prediction efficiency. As shown in Figure 2, the area under the curve (AUC) of overall survival (OS) was 0.764 (0.756~0.772) and cancer-specific survival (CSS) was 0.742 (0.733~0.751). In the validation group, the AUC of OS was 0.767 (0.689~0.846) and CSS was 0.742 (0.656~0.83). The calibration plots (Figure 3) and DCA analysis (Figure 4) revealed that the nomograms were good at elderly DLBCL patients' early death prediction and clinical application.

## 4. Discussion

DLBCL is an aggressive carcinoma and mainly affects the older population. Age is a negative prognostic factor for patients with DLBCL, which has been involved in the International Prognostic Index (IPI) [15, 16]. The prognosis of elderly DLBCL patients is worse than that of young patients [17]. Research revealed that the 5-year survival rate of young patients with DLBCL (≤55 years old) was 78%, which was only 54% among patients over 65 years old [18], illustrating the importance of predicting prognosis in elderly DLBCL patients. IPI contains five variables, including age, Ann Arbor stage, the number of extranodal sites, lactate dehydrogenase (LDH), and the Eastern Cooperative Oncology Group performance status [15]. At present, IPI is used as a guide for patients' survival and prognosis. However, IPI fails to identify patients at extreme risk [19, 20].

In this population-based research, we found that the overall early mortality of elderly DLBCL patients was 36.6% and 30.7% of them were DLBCL-specific early death, indicating the poor prognosis of elderly DLBCL patients, which consisted with the previous studies [16, 21].

The prognosis of the elderly DLBCL patients has always been a concern, and most studies are mainly focused on its long-term survival. In recent years, early death of DLBCL patients has attracted much attention. Cho et al. found that the patients suffering DLBCL with a survival time of less than 120 days accounted for 25%; meanwhile, old age, bone marrow involvement, and high-risk IPI score were risk factors for early mortality caused by DLBCL [22]. In 2016, Olszewski et al. quantified the risk factors for death and hospitalization within the first 30 days of rituximab-based immunochemotherapy [7]. We fully included clinical characteristic variables in this research, finding that marital status, Ann Arbor stage, surgical treatment, radiotherapy, and chemotherapy were significantly associated with the probability of early OS and CSS of elderly DLBCL patients. As a common clinical staging method for NHL, Ann Arbor staging is reasonably used as a predictor for elderly DLBCL patients.

The studies focused on early deaths have been applied to many types of carcinomas, which have shown an important clinical significance. Nomogram is a popular prognostic tool, which plays an important role in the identification of risk factors and personalized treatment [23, 24]. The research of IPI-DLBCL model is mainly focused on the medical indexes while ignoring the influence of families [25]. In this research, marital status, Ann Arbor stage, surgical treatment, radiotherapy, and chemotherapy were used to construct the predictive nomogram models. Therefore, this research may provide a new way to explore a better prognostic model. Moreover, as a dynamic model, nomogram plays an important role in disease heterogeneity and individualized therapy. In validation, we found that there was a good agreement between predicted early deaths and actual deaths. Besides that, DCA analysis revealed that our nomogram models had a good clinical value and utility in predicting survival.

The research has several strengths. Firstly, SEER database contains information of DLBCL patients, which involves about 27.8% of cancerous patients in the US; thus, the information is reliable. Secondly, external data is used for validation, which makes the predictive models more realistic and credible. However, our study also exists some limitations. For instance, SEER is a high-quality registry that collects clinically relevant features for risk prediction and does not collect the frailty characteristics and gene information of older individuals. Besides, the information of specific drugs of chemotherapy regimens is incomplete, and molecular pathological indicators are not involved in this research, which are also the limitation of our study.

## 5. Conclusion

In conclusion, marital status, Ann Arbor stage, surgical treatment, radiotherapy, and chemotherapy were risk factors for elderly DLBCL patients. Dynamic nomogram models were constructed to predict the early OS and CSS of elderly DLBCL patients, which might be beneficial to elderly DLBCL patients' early death prediction and clinical application.

## Figures and Tables

**Table 1:** Clinicopathological characteristics of patients in the SEER database and external cohort.

	Variable	SEER	External validation
*n*		15242	152

Sex (%)	Male	7485 (49.1)	77 (50.7)
	Female	7757 (50.9)	75 (49.3)

Race (%)	White	13122 (86.1)	NA
	Black	658 (4.3)	NA
	AI/AN	75 (0.5)	NA
	API	1387 (9.1)	NA

Marital status (%)	Married	8139 (53.4)	87 (57.2)
	Unmarried	7103 (46.6)	65 (42.8)

Median household income (%)	<$40,000	540 (3.5)	NA
	$40,000–$54,999	2892 (19.0)	NA
	$55,000–$69,999	5950 (39.0)	NA
	>$70,000	5860 (38.4)	NA

Tumor site (%)	Nodal	9784 (64.2)	96 (63.2)
	Extranodal	5458 (35.8)	56 (36.8)

Ann Arbor stage (%)	I	3829 (25.1)	33 (21.7)
	II	2710 (17.8)	26 (17.1)
	III	2572 (16.9)	28 (18.4)
	IV	5282 (34.7)	60 (39.5)
	Unknown	849 (5.6)	5 (3.3)

Surgery (%)	No	11027 (72.3)	111 (73.0)
	Yes	4215 (27.7)	41 (27.0)

Radiation (%)	No/unknown	12838 (84.2)	134 (88.2)
	Yes	2404 (15.8)	18 (11.8)

Chemotherapy (%)	No/unknown	5466 (35.9)	37 (24.3)
	Yes	9776 (64.1)	115 (75.7)

**Table 2:** Rate of early death in older patients with DLBCL.

	Variable	Overall	No early death	Total early death	Cancer-specific early death
*n*		15242	9658	5584	4680

Sex (%)	Male	7485 (49.1)	4724 (48.9)	2761 (49.4)	2263 (48.4)
	Female	7757 (50.9)	4934 (51.1)	2823 (50.6)	2417 (51.6)

Race (%)	White	13122 (86.1)	8320 (86.1)	4802 (86.0)	4016 (85.8)
	Black	658 (4.3)	406 (4.2)	252 (4.5)	217 (4.6)
	AI/AN	75 (0.5)	54 (0.6)	21 (0.4)	19 (0.4)
	API	1387 (9.1)	878 (9.1)	509 (9.1)	428 (9.1)

Marital status (%)	Married	8139 (53.4)	5423 (56.2)	2716 (48.6)	2266 (48.4)
	Unmarried	7103 (46.6)	4235 (43.8)	2868 (51.4)	2414 (51.6)

Median household income (%)	<$40,000	540 (3.5)	329 (3.4)	211 (3.8)	174 (3.7)
	$40,000–$54,999	2892 (19.0)	1800 (18.6)	1092 (19.6)	906 (19.4)
	$55,000–$69,999	5950 (39.0)	3714 (38.5)	2236 (40.0)	1892 (40.4)
	>$70,000	5860 (38.4)	3815 (39.5)	2045 (36.6)	1708 (36.5)

Tumor site (%)	Nodal	9784 (64.2)	6224 (64.4)	3560 (63.8)	3018 (64.5)
	Extranodal	5458 (35.8)	3434 (35.6)	2024 (36.2)	1662 (35.5)

Ann Arbor stage (%)	I	3829 (25.1)	2663 (27.6)	1166 (20.9)	925 (19.8)
	II	2710 (17.8)	1824 (18.9)	886 (15.9)	739 (15.8)
	III	2572 (16.9)	1703 (17.6)	869 (15.6)	733 (15.7)
	IV	5282 (34.7)	2951 (30.6)	2331 (41.7)	2018 (43.1)
	Unknown	849 (5.6)	517 (5.4)	332 (5.9)	265 (5.7)

Surgery (%)	No	11027 (72.3)	6735 (69.7)	4292 (76.9)	3621 (77.4)
	Yes	4215 (27.7)	2923 (30.3)	1292 (23.1)	1059 (22.6)

Radiation (%)	No/unknown	12838 (84.2)	7677 (79.5)	5161 (92.4)	4314 (92.2)
	Yes	2404 (15.8)	1981 (20.5)	423 (7.6)	366 (7.8)

Chemotherapy (%)	No/unknown	5466 (35.9)	2013 (20.8)	3453 (61.8)	2868 (61.3)
	Yes	9776 (64.1)	7645 (79.2)	2131 (38.2)	1812 (38.7)

**Table 3:** Univariate and multivariate logistic analyses of variables associated with risk of OS for older DLBCL patients.

Variable	Univariate analysis			Multivariate analysis		
	OR	95% CI	*P*	OR	95% CI	*P*
Sex						
Male						
Female	0.98	0.92-1.05	0.527			
Race						
White						
Black	1.08	0.92-1.26	0.377			
AI/AN	0.67	0.41-1.12	0.126			
API	1	0.9-1.13	0.94			
Marital status						
Married						
Unmarried	1.35	1.27-1.44	<0.001	1.1	1.02-1.18	0.017
Median household income						
< $40,000						
$40,000–$54,999	0.95	0.78-1.14	0.563			
$55,000–$69,999	0.94	0.78-1.12	0.493			
> $70,000	0.84	0.7-1	0.052			
Tumor site						
Nodal						
Extranodal	1.03	0.96-1.1	0.392			
Ann Arbor stage						
I						
II	1.11	1-1.23	0.054	1.26	1.12-1.42	<0.001
III	1.17	1.05-1.3	0.005	1.48	1.31-1.67	<0.001
IV	1.8	1.65-1.97	<0.001	2.17	1.96-2.4	<0.001
Unknown	1.47	1.26-1.71	<0.001	0.86	0.72-1.02	0.077
Surgery						
No						
Yes	0.69	0.64-0.75	<0.001	0.67	0.61-0.73	<0.001
Radiation						
No/unknown						
Yes	0.32	0.28-0.35	<0.001	0.33	0.29-0.37	<0.001
Chemotherapy						
No/unknown						
Yes	0.16	0.15-0.17	<0.001	0.14	0.13-0.15	<0.001

**Table 4:** Univariate and multivariate logistic analyses of variables associated with risk of CSS for older DLBCL patients.

Variable	Univariate analysis			Multivariate analysis		
	OR	95% CI	*P*	OR	95% CI	*P*
Sex						
Male						
Female	1.04	0.97-1.12	0.216			
Race						
White						
Black	1.12	0.94-1.32	0.198			
AI/AN	0.77	0.46-1.3	0.324			
API	1.01	0.9-1.14	0.846			
Marital status						
Married						
Unmarried	1.33	1.25-1.43	<0.001	1.11	1.03-1.19	0.009
Median household income						
< $40,000						
$40,000–$54,999	0.96	0.79-1.17	0.681			
$55,000–$69,999	0.98	0.81-1.18	0.84			
> $70,000	0.87	0.72-1.05	0.134			
Tumor size						
Nodal						
Extranodal	0.98	0.91-1.05	0.612			
Ann Arbor stage						
I						
II	1.18	1.05-1.32	0.004	1.32	1.16-1.49	<0.001
III	1.25	1.12-1.4	<0.001	1.54	1.36-1.75	<0.001
IV	1.94	1.77-2.13	<0.001	2.24	2.02-2.49	<0.001
Unknown	1.42	1.21-1.68	<0.001	0.89	0.75-1.07	0.216
Surgery						
No						
Yes	0.69	0.63-0.74	<0.001	0.69	0.63-0.75	<0.001
Radiation						
No/unknown						
Yes	0.35	0.32-0.4	<0.001	0.39	0.35-0.45	<0.001
Chemotherapy						
No/unknown						
Yes	0.21	0.19-0.22	<0.001	0.18	0.17-0.2	<0.001

## Data Availability

The datasets used and analyzed during the current study are available from the corresponding author on reasonable request.
